# Associations of job satisfaction and work-family conflict with turnover intention among nursing assistants in chinese long-term care: the mediating role of psychological health

**DOI:** 10.1186/s12913-026-14496-0

**Published:** 2026-05-04

**Authors:** Yiting Yu, Changxian Sun, Kai Qian, Sisi Wang, Yaping Ding, Xia Wan, Rui Liu

**Affiliations:** 1 School of Nursing, Xiamen Medical College, Xiamen, China; 2 College of Health, Jiangsu Vocational Institute of Commerce, Nanjing, China; 3 School of Nursing, Nanjing Medical University, No.101, Longmian Avenue, Jiangning District, Nanjing, China; 4 Department of Geriatrics, The Affiliated Wuxi People’s Hospital of Nanjing Medical, Wuxi, China; 5 Wuxi People’s Hospital, Wuxi, China; 6 Wuxi Medical Center, Nanjing Medical University, 299 Qingyang Road, Liangxi District, Wuxi, Jiangsu 214000 China; 7 School of Nursing and Health Management, Shanghai University of Medicine & Health Sciences, No.279, Zhouzhu Highway, Pudong New Area, Shanghai, 201813 China

**Keywords:** Job satisfaction, Work-family conflict, Psychological distress, Turnover intentions, Nursing assistants, Mediation

## Abstract

**Background:**

This study examines the relationships among job satisfaction, work-family conflict, psychological distress, and turnover intention among nursing assistants in China, with a particular focus on the mediating role of psychological distress.

**Methods:**

A cross-sectional survey was conducted among 240 nursing assistants from Jiangsu and Fujian provinces between December 2023 and March 2024. Standardized instruments measured job satisfaction, work-family conflict, psychological distress (K10), and turnover intention. Pearson correlation and mediation analyses were performed using PROCESS Macro (Model 4) to assess relationships and mediating effects.

**Results:**

Job satisfaction was negatively associated with turnover intention (*r*=-0.30, *p* < 0.01) and psychological distress (*r*=-0.23, *p* < 0.01). Work-family conflict was positively correlated with psychological distress (*r* = 0.52, *p* < 0.01) and turnover intention (*r* = 0.48, *p* < 0.01). Mediation analyses indicated that psychological distress partially explained the associations of job satisfaction and work-family conflict with turnover intention, accounting for 17.64% and 27.22% of the total effects, respectively.

**Conclusion:**

Job satisfaction and work-family conflict demonstrate both direct associations with turnover intention and indirect associations through psychological distress. These findings suggest that integrating psychological health protection into workforce management strategies, alongside reducing work-family conflict and strengthening organizational resources, may represent a viable approach to improving retention in China’s long-term care sector.

**Clinical trial number:**

Not applicable.

## Background

The accelerating global aging trend has increased the demand for long-term care services, creating an urgent need for a stable caregiving workforce. Yet persistent shortages and high turnover rates among nursing assistants remain critical challenges worldwide. In Japan, only 40.1% of nursing staff intend to remain in their current roles [1], while in Germany, nearly one-third consider leaving within a year [2]. In China, the situation is even more severe. Projections estimate that by 2050, at least 33 million nursing assistants will be needed, although fewer than one million are currently employed [3, 4]. Turnover rates remain high (30–40%) [5], and dropout rates among elderly care students approach 70% [6]. Collectively, these trends signal not only a severe workforce shortage but also an urgent need to understand the psychological and work-related mechanisms driving turnover intention among nursing assistants, particularly within long-term care settings.

Turnover intention, widely regarded as a proximal predictor of actual resignation, imposes financial and operational burdens on care facilities [7, 8]. Previous studies have identified job satisfaction, work-family conflict, and psychological health as key contributors to turnover intention [9–12]. Even in countries like South Korea and the United States, where policies aim to improve retention, caregivers continue to report heavy workloads, emotional demands, and limited career advancement [13, 14]. Nursing assistants with poorer psychological health, higher work-family conflict, and lower job satisfaction consistently report stronger turnover intentions [15]. Therefore, these factors do not operate in isolation; rather, they interact through psychological processes whereby adverse work conditions undermine mental well-being and shape withdrawal cognitions such as turnover intention.

Job satisfaction is a central factor influencing caregiver retention. Across studies conducted in China, Japan, and the United States, dissatisfaction is commonly linked to long working hours, low wages, and limited development opportunities [16–18]. In China, over 50% of caregivers report dissatisfaction with their jobs, often due to low pay, heavy workloads, and insufficient organizational support [19]. Within the JD-R framework, job satisfaction can be conceptualized as a key job-related resource that enhances psychological well-being, whereas persistent dissatisfaction signals resource insufficiency and increases vulnerability to stress, burnout, and withdrawal from the profession.

Work-family conflict is another major contributor to turnover. It reflects the competing pressures of work and family roles and is especially prominent among nursing assistants, who often face inflexible schedules and long shifts [20, 21]. Research from both Western and East Asian contexts shows that work-family conflict is widespread [22–24]. In collectivist cultures such as China and Korea, where family obligations are strongly emphasized, this conflict may be more emotionally demanding [25–27].

Psychological health, defined as emotional and mental well-being, is increasingly conceptualized not merely as an outcome of work stressors but as a key psychological pathway linking adverse work conditions to turnover-related behaviors [28, 29]. In this study, psychological health is operationalized and measured as psychological distress. Although traditional occupational models frequently utilize burnout as the primary strain indicator, burnout is largely domain-specific to the work environment. In contrast, work-family conflict is a boundary-spanning stressor whose adverse effects spill over into personal life [30]. Therefore, a generalized measure of psychological distress more accurately captures the broad emotional toll of these cross-domain pressures than conventional burnout scales. Elevated psychological distress is associated with stress, anxiety, and burnout, which contribute to disengagement and turnover intention [31, 32]. In China, more than 75% of caregivers experiencing high work-family conflict report symptoms of severe psychological distress [33]. Conversely, psychological resilience appears to buffer against job dissatisfaction and turnover intention [34].

To explain how job satisfaction and work-family conflict shape turnover intention, this study draws on the Job Demands-Resources (JD-R) model and the Conservation of Resources (COR) theory. From a JD-R perspective, work-family conflict represents a salient job demand that requires sustained emotional and cognitive effort, increasing the risk of psychological strain through the health impairment process. Such psychological distress has been consistently linked to withdrawal cognitions, including turnover intention [35, 36]. According to the dual-process logic of the JD-R model, excessive job demands consume physical and psychological energy, leading to impaired well-being and subsequent withdrawal-related cognitions [37].

In contrast, job satisfaction reflects a key job-related resource that supports psychological well-being. According to COR theory, individuals strive to conserve valued resources such as time, energy, and emotional stability [38], when these resources are persistently threatened, withdrawal from the resource-depleting environment becomes a rational coping response. Job satisfaction thus acts as a proxy for resource sufficiency, limited resource gain heightens vulnerability to psychological strain and subsequent turnover intention [39]. Accordingly, psychological distress is conceptualized as a central mechanism linking job demands and job resources to turnover intention. This sequence aligns perfectly with the environment-strain-behavior paradigm, illustrating how external occupational factors shape internal psychological states and ultimately drive behavioral cognition [37, 39].

However, the mechanisms of the JD-R and COR models may not operate uniformly across different institutional and cultural settings. The Chinese long-term care (LTC) sector presents a structurally unique environment currently undergoing rapid expansion and policy evolution. As the LTC system navigates institutional consolidation through the pilot Long-Term Care Insurance (LTCI) scheme [40], transitional challenges such as organizational instability, severe staffing shortages, and limited professionalization become unavoidable. These systemic pressures inevitably amplify perceived job demands and lower the threshold for resource depletion [12], rendering the health impairment process highly salient among frontline caregivers.

Moreover, caregiving in China is culturally embedded within Confucian norms of filial piety (Xiao), where eldercare is traditionally regarded as a sacred family responsibility [41]. When such care is transferred to institutional workers, nursing assistants often experience an “ambiguous social status”, performing culturally valued work while occupying relatively low-prestige occupational positions [42]. In this collectivist context where family obligations are normatively emphasized, work-family conflict may carry stronger emotional and moral implications, thereby amplifying its impact on psychological distress [43].

Despite the growing application of these theories, empirical studies examining their integrated mechanisms within this unique Chinese occupational and cultural context remain scarce. Given the persistently high turnover in China, a clearer understanding of these pathways is urgently needed. The present study examines the mediating role of psychological distress in the relationships among job satisfaction, work-family conflict, and turnover intention among nursing assistants in Chinese long-term care settings, and proposes the following hypotheses based on the established causal ordering:

## Direct effects on turnover intention

Based on COR theory, job dissatisfaction represents a sustained resource deficit, prompting withdrawal behaviors to prevent further emotional depletion. Concurrently, within the JD-R framework, work-family conflict acts as an acute job demand, when these pressures exceed coping capacities, the intention to leave emerges as a primary mitigating strategy. Therefore, we hypothesize:

### H1

 Job satisfaction is negatively associated with turnover intention.

### H2

Work-family conflict is positively associated with turnover intention.

### The mediating role of psychological distress

Beyond these direct effects, our model aligns with the environment-strain-behavior paradigm, positing that workplace conditions influence withdrawal cognitions through an internal psychological pathway. Specifically, the combined energy drain from low resources and high demands severely exacerbates psychological distress, which subsequently drives the intention to resign. Accordingly, we hypothesize:

#### H3

Psychological distress mediates the relationship between job satisfaction and turnover intention.

#### H4

Psychological distress mediates the relationship between work-family conflict and turnover intention.

**Fig. 1:**
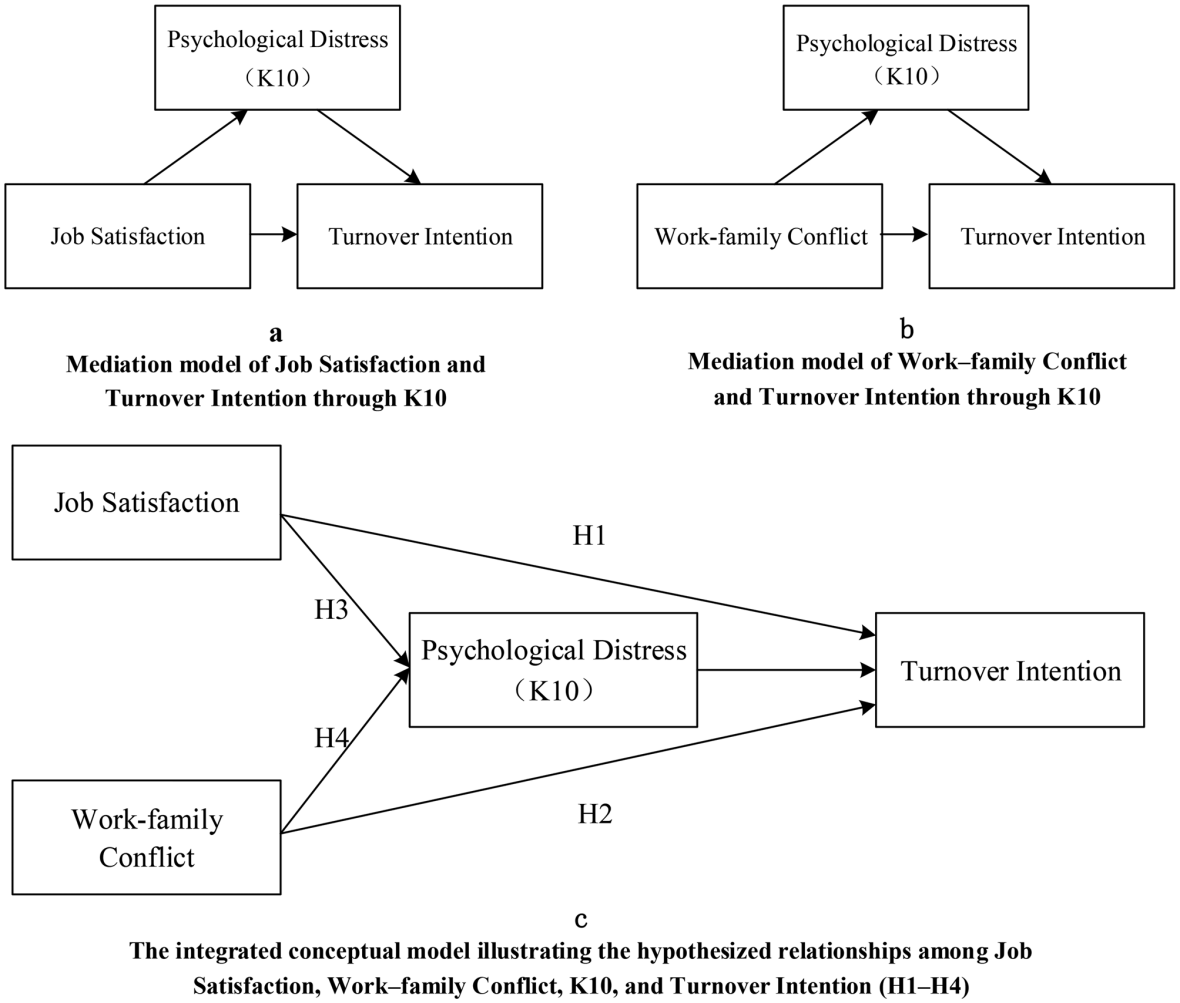
The conceptual mediation model relating Job Satisfaction, Work-family Conflict, K10, and Turnover Intention. *K10* the 10-item kessler psychological distress scale

The conceptual framework underlying these hypotheses is presented in Fig. 1

## Methods

### Ethical considerations

This study was approved by the Ethics Committee of Nanjing Medical University (No. (2021)605). Prior to data collection, all participants were fully informed about the study’s objectives and procedures. Participation was voluntary, and individuals had the right to withdraw at any point without penalty. Informed consent was obtained from all participants prior to their involvement in the research. The study was conducted in accordance with the ethical principles of the Declaration of Helsinki.

### Settings and samples

A cross-sectional design with convenience sampling was employed to recruit participants from nursing homes across urban and rural regions in eastern China. Inclusion criteria were: (1) aged 18 years or older; (2) possession of a caregiver certificate and a minimum of one month of work experience; and (3) direct involvement in elderly care. Exclusion criteria included cognitive or physical difficulties that interfered with survey completion or lack of caregiving responsibilities.

An a priori power analysis was conducted using G*Power 3.1 to determine the required sample size for multiple linear regression analysis (two predictors, α = 0.05, power = 0.80, medium effect size f²=0.15), which indicated a minimum sample size of 114 participants [44]. To account for potential incomplete responses and enhance the robustness of mediation analysis using bootstrapping procedures [45], a target sample size of at least 200 was set. Ultimately, 240 valid questionnaires were collected, exceeding the minimum requirement and supporting the adequacy of the sample for the planned analyses.

### Data collection

Data were collected from December 2023 and March 2024 using Wenjuanxing (https://www.wjx.cn/), a secure and widely-used online survey platform in China. The research team underwent training to ensure consistent implementation of ethical and procedural standards. Prior to the full launch, a pilot test was conducted to ensure clarity of items and platform usability.

Participants accessed the survey via a hyperlink or QR code shared through social media platforms. An online consent form preceded the questionnaire to ensure informed participation. Each IP address and account was restricted to a single submission to prevent duplication. Real-time data backups were enabled to avoid data loss. The estimated time to complete the questionnaire was 10–15 min.

### Measurement

A structured questionnaire was used comprising the following instruments:

#### Demographic questionnaire

The demographic characteristics included gender, age, educational background, marital status, work experience, nature of employment, working shift, monthly income, workplace location, nursing work object, number of children, living conditions, and whether participants had received formal nursing education.

#### Job satisfaction survey (JSS)

Developed by Schreisheim and Tsui [46], this 6-item scale measures satisfaction with various job aspects using a 5-point Likert scale (1 = strongly disagree to 5 = strongly agree). The total score is computed by averaging all item scores, with higher scores indicating greater job satisfaction. The Cronbach’s α coefficient for the Job Satisfaction Survey is 0.90 [47]. In this study, the Cronbach’s α coefficient for the scale is 0.91. The scale is openly accessible for use at https://scales.arabpsychology.com/s/job-satisfaction-index/.

#### The chinese version of the work-family balance scale (WFCS)

Adapted from Grzywacz and Marks [48], and translated into Chinese by Zeng [49], this 14-item scale includes four subdimensions: work-family conflict (4 items), work-family facilitation (3 items), family-work conflict (4 items), and family-work facilitation (3 items). The scale is further divided into two main aspects: work-family conflict (8 items) and work-family facilitation (6 items). Responses are measured using a 5 = point Likert scale (1 = strongly disagree to 5 = strongly agree). The retest reliability of the Chinese version for both the total scale and each subscale exceeds 0.75, and the internal consistency (Cronbach’s α) is above 0.68 [47]. In this study, The Cronbach’s α coefficients of each dimension in this study were 0.86, 0.80, 0.91 and 0.81, respectively. Cronbach’s α coefficient of total quantity table is 0.89.

#### The chinese version of psychological distress scale (K10)

Developed by Kessler [50], this 10-item scale assesses levels of stress, anxiety, and depression experienced over the past month. Items are rated from 1 (none of the time) to 5 (all of the time). Higher total scores indicate higher levels of psychological distress. The total score is the sum of the items, ranging from 10 to 50. The Chinese version has demonstrated good psychometric properties, Cronbach’s α was 0.80 [51]. In this study, Cronbach’s α was 0.96. The scale is openly accessible for use at https://www.tac.vic.gov.au/files-to-move/media/upload/k10_english.pdf.

#### The chinese version of the turnover intention scale (TIS)

Developed by Farh [52], this 4-item scale assesses the likelihood of employees leaving their organization. It uses a 5-point Likert scale, with item three reverse-coded. Higher total scores (range: 4–20) reflect greater turnover intention. The Chinese version shows high validity and reliability, and the internal consistency reliability is 0.84 [53]. The Cronbach’s α coefficient of the scale was 0.93, in this study and the split-half reliability was 0.92.

#### Control variables

To minimize confounding, demographic variables known to influence turnover intention were included as controls in the regression analyses, including age, gender, educational background, marital status, work experience, nature of employment, monthly income, workplace location, number of children and received nursing formal education.

### Data analysis

Data were analyzed using SPSS 23.0. Descriptive statistics summarized demographic characteristics. For continuous variables (e.g., work experience), medians and interquartile ranges (IQRs) were reported; categorical variables were expressed as frequencies and percentages. Group differences in turnover intention across demographic subgroups were assessed using the Kruskal-Wallis test. Pearson correlation analysis examined associations among job satisfaction, work-family conflict, psychological distress, and turnover intention.

To test the mediation model, PROCESS macro (version 3.3) by Hayes [54] was used (Model 4). Bootstrapping (5,000 samples) was conducted to estimate the indirect effect of psychological distress. Mediation was considered statistically significant if the 95% confidence interval (CI) did not include zero. The proportion mediated was calculated as the indirect effect divided by the total effect. To ensure consistency and comparability across analyses, all regression and mediation models were adjusted for the same set of demographic covariates described in the Control Variables section. Given the cross-sectional study design, the mediation analyses were interpreted as statistical associations rather than evidence of causal relationships.

## Result

### Demographic characteristics of study participants

A total of 247 nursing assistants were recruited, and 240 valid questionnaires were returned (response rate: 97.17%). Participants were aged 18–60 years and had a median of 4 years of work experience (IQR: 1–7 years). More than half (54.58%) had less than 5 years of experience. The sample was predominantly female (76.67%) and married (58.75%). Most participants worked full-time (85.83%), and over half were on shift schedules. The majority were employed in urban facilities (85.00%). Regarding care recipients, 67.08% provided care for older adults with functional independence. Participants had an average of one child (range 0–2), and 53.33% lived with family members. In addition, 80% had received formal nursing-related education.

Mean scores for main variables were: work-family conflict (M = 2.78, SD = 0.96), job satisfaction (M = 3.60, SD = 0.89), psychological distress (M = 23.41, SD = 8.99), and turnover intention (M = 10.21, SD = 4.37). Detailed participant characteristics are presented in Table 1.

### Descriptive statistics and correlations among core variables

Work-family conflict differed significantly across educational background (H = 15.10, *p* < 0.01), monthly income (H = 8.54, *p* = 0.04), and number of children (H = 15.17, *p* < 0.01). Job satisfaction varied by educational background (H = 15.86, *p* < 0.01), marital status (H = 10.88, *p* = 0.01), work experience (H = 6.26, *p* = 0.04), monthly income (H = 8.69, *p* = 0.03), and receipt of nursing formal education (H = 3.86, *p* = 0.05).

Psychological distress was significantly associated with gender (H = 5.85, *p* = 0.02), age (H = 24.40, *p* < 0.01), educational background (H = 28.80, *p* < 0.01), marital status (H = 14.41, *p* < 0.01), work experience (H = 8.04, *p* = 0.02), employment type (H = 8.52, *p* < 0.01), monthly income (H = 24.68, *p* < 0.01), number of children (H = 17.84, *p* < 0.01), and nursing formal education (H = 6.66, *p* = 0.01). Turnover intention also varied across most of these variables and workplace location (H = 6.63, *p* = 0.04).

Pearson correlations (Table 2) showed that job satisfaction was negatively correlated with psychological distress (*r*=-0.23, *p* < 0.01) and turnover intention (*r*=-0.30, *p* < 0.01). Work-family conflict was positively correlated with psychological distress (*r* = 0.52, *p* < 0.01) and turnover intention (*r* = 0.48, *p* < 0.01). Psychological distress was also positively correlated with turnover intention (*r* = 0.52, *p* < 0.01). To address potential conceptual overlap among the constructs, collinearity diagnostics were performed. The Variance Inflation Factor (VIF) values for all predictors were well below the stringent threshold of 3.0, indicating that multicollinearity was not a concern. In addition, Harman’s single-factor test was conducted to assess potential common method bias. The results of the unrotated exploratory factor analysis showed that the first factor accounted for 31.74% of the total variance, which is well below the widely accepted 50% threshold [55]. This suggests that common method bias is unlikely to significantly affect our findings.

### Mediating effects of psychological distress

Table 3 presents the mediation analysis using Hayes’ PROCESS Model 4. In Model 1, work-family conflict (B = 1.80, t = 7.16, *p* < 0.01) and job satisfaction (B=-1.19, t=-4.37, *p* < 0.01) were significant predictors of turnover intention. In Model 2, work-family conflict (B = 3.96, t = 7.95, *p* < 0.01) was positively associated with psychological distress, while job satisfaction negatively predicted psychological distress (B=-1.70, t=-3.15, *p* < 0.01).

In Model 3, psychological distress significantly predicted turnover intention (B = 0.13, t = 3.82, *p* < 0.01), After adding psychological distress to the model, work-family conflict (B = 1.31, t = 4.73, *p* < 0.01) and job satisfaction (B=-0.98, t=-3.62, *p* < 0.01) remained significant, indicating partial mediation. All models controlled for gender, age, marital status, educational background, work experience, and income.

Table 4 displays the bootstrapping results for indirect effects. The 95% confidence intervals did not include zero, confirming significant mediation. Work-family conflict and job satisfaction directly predicted turnover intention (direct effects: 1.80 and − 1.19, respectively), also had indirect effects via psychological distress (indirect effects: 0.49 and − 0.21). The mediating effect accounted for 27.22% and 17.64% of the total effects, respectively (total effects: 1.31 for work-family conflict, -0.98 for job satisfaction).

**Table 1:** Distribution of characteristics of participants and the differences among work-family conflict, job satisfaction and psychological distress (*N* = 240)

Characteristics category	*N*(%)	Median(IQR)	Work-family Conflict				Job Satisfaction				Psychological Distress				Turnover Intention			
			Median	SD	H	*P*	Median	SD	H	*P*	Median	SD	H	*P*	Median	SD	H	*P*
Gender																		
Male	56(23.33)		2.94	0.95	2.94	0.09	3.42	0.91	3.42	0.06	25.00	9.33	5.85	**0.02**	12.00	4.39	4.71	**0.03**
Female	184(76.67)		2.75	0.96			3.67	0.88			22.00	8.74			10.00	4.31		
Age(year)																		
18–30	89(37.08)		2.88	0.95	6.86	0.08	3.50	0.84	2.78	0.43	28.00	9.24	24.40	**0.00**	12.00	3.85	29.18	**0.00**
31–40	45(18.75)		2.88	1.07			3.67	0.83			21.00	8.08			9.00	4.12		
41–50	44(18.33)		2.50	0.77			3.67	0.96			21.50	7.59			8.50	4.20		
51–60	62(25.84)		2.63	0.97			3.83	0.96			20.00	8.45			8.00	4.49		
Educational background																		
Primary school and below	15(6.25)		2.75	0.92	15.10	**0.00**	3.67	0.87	15.86	**0.00**	20.00	9.08	28.80	**0.00**	8.00	4.87	17.91	**0.00**
Junior high school	49(20.42)		2.63	0.96			3.67	0.99			21.00	8.11			9.00	4.27		
High school	55(22.92)		2.50	0.94			4.00	0.76			18.00	8.38			8.00	4.47		
University and above	121(50.41)		3.00	0.93			3.50	0.86			27.00	8.74			12.00	4.05		
Marital status																		
Unmarried	80(33.34)		2.88	0.92	6.67	0.08	3.50	0.85	10.88	**0.01**	26.00	8.12	14.41	**0.00**	12.00	3.64	24.08	**0.00**
Married	141(58.75)		2.63	0.93			3.67	0.87			21.00	8.23			9.00	4.07		
Divorce	14(5.83)		2.94	0.98			4.08	1.12			23.00	11.65			11.50	6.16		
Widowed	5(2.08)		3.00	1.67			5.00	0.89			30.00	19.28			12.00	8.00		
Work experience (year)		4(1,7)																
<5	131(54.58)		2.75	0.93	2.20	0.33	3.67	0.89	6.26	**0.04**	24.00	9.39	8.04	**0.02**	12.00	4.29	15.53	**0.00**
5–10	90(37.50)		2.88	0.98			3.50	0.89			22.00	7.82			9.50	4.14		
>10	19(7.92)		2.38	0.98			4.00	0.79			13.00	9.82			6.00	4.43		
Nature of employment																		
Full-time	206(85.83)		2.75	0.96	0.48	0.49	3.67	0.89	0.04	0.85	22.00	8.95	8.52	**0.00**	10.00	4.41	5.20	**0.02**
Part-time job	34(14.17)		2.88	0.93			3.92	0.91			28.50	8.21			12.00	3.84		
Working shift																		
White shift	100(41.67)		2.69	1.01	2.60	0.27	3.67	0.84	0.81	0.67	22.00	9.03	0.67	0.72	11.00	4.36	2.98	0.23
Night shift	15(6.25)		2.25	0.89			3.67	1.02			21.00	8.97			12.00	4.72		
Shifting	125(52.08)		2.88	0.92			3.50	0.92			23.00	9.01			10.00	4.32		
Monthly income (RMB)																		
< 3000	33(13.75)		2.88	1.04			4.00	0.85			30.00	10.58			12.00	4.53		
3000–4000	40(16.67)		2.94	0.82	8.54	**0.04**	3.42	0.80	8.69	**0.03**	28.00	7.89	24.68	**0.00**	12.00	3.77	17.55	**0.00**
4001–5000	66(27.50)		3.00	0.91			3.50	0.94			23.00	7.87			10.00	4.35		
> 5000	101(42.08)		2.50	0.98			3.83	0.89			19.00	8.50			9.00	4.26		
Workplace location																		
City	204(85.00)		2.75	0.94	5.18	0.08	3.67	0.91	1.20	0.55	22.00	8.61	5.57	0.06	10.00	4.36	6.63	**0.04**
Rural	23(9.58)		3.00	1.02			3.67	0.80			30.00	10.71			12.00	4.08		
Suburbs	13(5.42)		3.13	0.93			3.50	0.81			28.00	9.95			10.00	4.23		
Nursing work object																		
Self-care elderly	161(67.08)		2.75	1.03	0.25	0.62	3.67	0.89	2.77	0.10	23.00	9.00	0.12	0.73	10.00	4.39	1.29	0.26
Semi-self-care elderly	79(32.92)		2.88	0.80			3.50	0.87			23.00	9.00			10.00	4.32		
Number of children		1(0,2)																
0	91(37.92)		2.88	0.90	15.71	**0.00**	3.50	0.86	2.70	0.44	26.00	8.75	17.84	**0.00**	12.00	3.89	21.88	**0.00**
1	68(28.33)		2.50	0.90			3.67	0.93			20.50	8.15			9.00	4.59		
2	69(28.75)		2.63	0.97			3.67	0.93			21.00	8.72			8.00	3.82		
3	12(5.00)		3.63	1.05			4.00	0.74			21.50	10.93			10.00	6.05		
Living conditions																		
Alone	38(15.83)		3.00	1.05	3.48	0.18	3.50	0.98	4.06	0.13	23.00	10.66	1.20	0.55	11.50	5.18	3.66	0.16
With your family	128(53.33)		2.75	1.05			3.67	0.90			22.00	9.09			10.00	4.51		
With colleagues/friends	74(30.84)		2.75	0.71			3.50	0.82			23.00	7.77			10.00	3.52		
Received nursing formal education																		
YES	192(80.00)		2.75	0.98	2.06	0.15	3.67	0.91	3.86	**0.05**	22.00	8.71	6.66	**0.01**	10.00	4.33	3.80	**0.05**
NO	48(20.00)		2.94	0.83			3.50	0.81			26.00	9.45			11.50	4.40		

**Table 2:** Correlation matrix for job satisfaction, work-family conflict, psychological distress and turnover intention (*N* = 240)

	Job Satisfaction	Work-family Conflict	Psychological Distress	Turnover Intention
	Job Satisfaction	1		
Work-family Conflict	-0.09	1		
Psychological Distress	-0.23^**^	0.52^**^	1	
Turnover Intention	-0.30^**^	0.48^**^	0.52^**^	1

**P < 0.01

**Table 3:** Regression coefficients for the mediation effect model (*N* = 240)

	Turnover Intention (Model 1)					Psychological Distress (Model 2)					Turnover Intention (Model 3)				
	Coefficient (B)	Standard Error (SE)	t	*P*	Standardized Coefficient (Beta)	Coefficient (B)	Standard Error (SE)	t	*P*	Standardized Coefficient (Beta)	Coefficient (B)	Standard Error (SE)	t	*P*	Standardized Coefficient (Beta)
Predictor variable	11.99	2.99	4.01	0.00	-	16.82	5.91	2.85	0.01	-	9.89	2.95	3.35	0.00	-
Work-family Conflict	**1.80**	**0.25**	**7.16**	**0.00**	0.40	**3.96**	**0.50**	**7.95**	**0.00**	0.42	1.31	**0.28**	**4.73**	**0.00**	0.29
Job Satisfaction	**-1.19**	**0.27**	**-4.37**	**0.00**	-0.24	**-1.70**	**0.54**	**-3.15**	**0.00**	-0.17	-0.98	**0.27**	**-3.62**	**0.00**	-0.20
Gender	-0.58	0.57	-1.02	0.31	-0.06	-1.88	1.13	-1.66	0.10	-0.09	-0.35	0.56	-0.63	0.53	-0.03
Age	-0.57	0.33	-1.72	0.09	-0.16	-0.97	0.66	-1.47	0.14	-0.13	-0.45	0.33	-1.39	0.17	-0.13
Marital status	0.72	0.51	1.42	0.16	0.11	1.77	1.00	1.77	0.08	0.13	0.50	0.50	1.01	0.31	0.07
Educational background	-0.07	0.40	-0.17	0.87	-0.02	0.81	0.79	1.02	0.31	0.09	-0.17	0.39	-0.43	0.66	-0.04
Work experience	-0.91	0.42	-2.14	0.03	-0.13	-0.24	0.84	-0.29	0.78	-0.02	-0.88	0.41	-2.13	0.04	-0.13
Monthly income	-0.17	0.26	-0.64	0.52	-0.04	-1.12	0.51	-2.21	0.03	-0.13	-0.03	0.25	-0.10	0.92	-0.01
Nature of employment	0.49	0.74	0.66	0.51	0.04	1.80	1.46	1.23	0.22	0.07	0.26	0.72	0.36	0.72	0.02
Workplace location	-0.05	0.47	-0.11	0.92	-0.01	0.59	0.94	0.63	0.53	0.03	-0.12	0.46	-0.27	0.79	-0.02
Number of children	-0.37	0.34	-1.09	0.28	-0.08	-0.35	0.68	-0.52	0.60	-0.04	-0.33	0.33	-0.99	0.32	-0.07
Received nursing formal education	0.47	0.64	0.75	0.46	0.04	2.40	1.26	1.91	0.06	0.11	0.18	0.62	0.28	0.78	0.02
Psychological Distress											**0.13**	**0.03**	**3.82**	**0.00**	0.26
R²	0.36					0.41					0.40				
Adjusted R²	0.33					0.38					0.37				
F	F(12, 227) = 10.84, *P* = 0.00					F(12, 227) = 13.23, *P* = 0.00					F(13, 226) = 11.73, *P* = 0.00				

Note: All regression models (Model 1, Model 2, and Model 3) were adjusted for the same set of demographic covariates listed in this table

**Table 4:** Summary of mediation effect test (*N* = 240)

Item	c Total Effect	a	a (*p*-value)	b	b (*p*-value)	a*b Mediating Effect	a*b (Boot SE)	a*b (z-value)	a*b (*p*-value)	a*b (95%BootCI)	c’ Direct Effect	c’ (*p*-value)	Relative effect value
Work-family Conflict → Psychological Distress → Turnover Intention	1.80	3.96	0.00	0.13	0.00	0.49	0.21	2.32	0.02	0.15 -1.00	1.31	0.00	27.22%
Job Satisfaction → Psychological Distress → Turnover Intention	-1.19	-1.70	0.00	0.13	0.00	-0.21	0.09	-2.28	0.02	-0.46 - -0.07	-0.98	0.00	17.64%

## Discussion

From a health services management perspective, this study extends the application of the Job Demands-Resources (JD-R) model and Conservation of Resources (COR) theory to long-term care settings by elucidating how frontline workforce conditions are statistically linked to turnover intention through psychological distress. Specifically, work-family conflict functioned as a salient job demand that activated the health impairment pathway, whereas job satisfaction operated as a critical job-related resource associated with better psychological well-being and lower turnover intention. These findings position the mitigation of psychological distress as a strategic lever through which organizational management practices may influence workforce stability in long-term care services.

### Addressing high turnover intentions in high-risk subgroups

This study examined the relationships among job satisfaction, work-family conflict, psychological distress, and turnover intentions among nursing assistants in China. The findings corroborate prior research indicating that psychological distress serves as an explanatory pathway linking work-family conflict and job satisfaction with turnover intention [56]. In the present study, psychological distress accounted for 27.22% and 17.64% of the total effects, respectively. These results offer practical guidance for administrators aiming to stabilize the long-term care workforce.

Turnover intention was especially elevated among female, younger (18–30 years), rural-based, and university-educated nursing assistants. These groups face multiple pressures, including cultural expectations regarding caregiving, limited career development, and reduced access to support, which intensify work-family conflict and psychological strain [57]. To reduce turnover in these populations, managers should implement targeted strategies. Structured mentorship programs, continuing education, and credentialing opportunities tailored to long-term care can mitigate career stagnation and strengthen professional identity [58]. Improving infrastructure and social support in rural settings, through relocation stipends, enhanced living facilities, and local community networks which can help reduce environmental stressors [59]. Nursing education should embed sector-specific career planning to align graduates’ expectations with the practical realities of elder care, thereby reducing mismatch-induced dissatisfaction [60].

### Operationalizing organizational support to alleviate work-family conflict

From a health services management perspective, these findings suggest that investments in psychological well-being should be viewed as strategic workforce interventions rather than ancillary welfare initiatives, with direct implications for staff retention and service continuity. Implementing flexible scheduling systems, such as allowing employees to select preferred shifts, adopt compressed workweeks, or pursue part-time roles, can help staff effectively balance their familial obligations. In addition, offering subsidies for temporary childcare or eldercare services and establishing transparent, performance-linked promotion systems can mitigate financial strain and foster a sense of organizational commitment [61]. These strategies are consistent with the COR theory, which posits that stress arises from the loss of personal resources, and that reinforcing or replenishing such resources (e.g., time, energy, emotional security) can prevent burnout and reduce turnover [35]. Importantly, workforce instability in long-term care settings not only affects staff outcomes but also undermines continuity of care and service quality, with broader implications for patient experience and system sustainability.

### Institutionalizing psychological health protection in care settings

To fully contextualize the mediating role of psychological distress, it is necessary to clarify its strong correlation with turnover intention (*r* = 0.52). Despite this empirical overlap, the two constructs represent distinct phases of the withdrawal process [7]. Psychological distress, measured via the K10 scale, reflects an affective state of generalized emotional depletion. In contrast, turnover intention constitutes a specific, forward-looking behavioral cognition. The direct progression from emotional strain to withdrawal intentions in our sample reflects a systemic vulnerability within Chinese long-term care settings. Because frontline nursing assistants typically lack access to formal psychological counseling or adequate organizational support, unmitigated distress is rarely buffered [40]. Consequently, when the cumulative weight of occupational and family demands compromises their mental well-being, resigning frequently emerges as the most immediate and viable coping strategy to prevent further emotional exhaustion.

Given the mediating role of psychological distress, organizations should establish systematic mental health support. Beyond raising general awareness, care institutions should embed Employee Assistance Programs (EAPs) that offer access to psychological counseling, crisis intervention services, and referral pathways for specialized care. Routine mental health screening should be integrated into occupational health practice, and supervisors should receive training to identify early signs of distress and respond appropriately. Interventions such as mindfulness programs and peer-support groups may enhance resilience and coping capacity [62, 63]. These measures foster a climate of psychological safety within the workforce while enhancing institutional performance and care continuity.

### Applying the job demands-resources model to optimize workplace conditions

The findings of this study directly corroborate the JD-R model’s core premise: that excessive job demands in the absence of adequate resources lead to adverse outcomes. In the context of long-term care, the high emotional and physical demands of caregiving are frequently exacerbated by understaffing and rigid schedules [64], creating a classic JD-R imbalance. To counteract this, management interventions must systematically address both sides of the equation. Regular workload assessments, adequate staffing, and collaborative teamwork can help reduce operational pressures. Enhancing job autonomy, expanding opportunities for professional development, and recognizing staff contributions may further strengthen engagement and job satisfaction [65].

### Economic justification for investing in workforce stability

From an economic standpoint, enhancing staff retention through targeted organizational interventions constitutes a prudent investment. High turnover entails substantial direct and indirect costs, including recruitment, training, lower initial productivity, and diminished continuity of care. In contrast, strategies that improve job satisfaction and organizational commitment yield measurable benefits. For instance, in Taiwan, an empowering leadership training intervention using a flipped learning approach improved nursing staff’s job well-being in long-term care facilities [66]. Adopting similar approaches in nursing homes could enhance workforce stability, resident care outcomes, and long-term financial sustainability.

### Practical implications for nursing management

This study advances the literature on caregiver retention by elucidating modifiable psychosocial and organizational pathways to turnover. By focusing on high-risk subgroups, operationalizing flexible workplace policies, embedding psychological support into organizational systems, and applying established theoretical models such as COR and JD-R, nursing administrators can build more resilient, engaged, and stable care teams within long-term care settings. These strategies not only safeguard employee well-being but also optimize organizational performance and care quality.

## Limitations

This study has several limitations that should be considered when interpreting the findings. Due to the cross-sectional nature of the data, the observed mediation effects represent statistical associations rather than causal relationships. Future longitudinal studies are needed to establish the temporal ordering of these variables. In addition, participants were recruited through convenience sampling from two provinces, which may limit the representativeness of the national nursing assistant workforce and warrants caution when generalizing the results to other regions or care settings. The use of an online survey may also have introduced response and self-selection bias, as individuals with greater digital access or stronger motivation to participate may have been more likely to respond. Furthermore, institutional clustering was not explicitly modeled, and unmeasured organizational characteristics may have contributed to the observed associations. All variables were assessed through self-report measures, which may increase the risk of common-method bias despite the use of validated instruments. Future research employing longitudinal, multi-source, and multilevel designs would help strengthen causal inference and provide a more comprehensive understanding of caregiver retention processes.

## Conclusions

Psychological distress plays a central role in shaping turnover intention among nursing assistants, serving as a key pathway through which work-family conflict and low job satisfaction are associated with intentions to leave. This mechanism appears particularly pronounced among high-risk subgroups, underscoring the importance of targeted workforce interventions. The findings suggest that reducing work-family conflict, strengthening organizational and psychological support, and optimizing working conditions in accordance with the JD-R model are integral to effective retention strategies. Beyond improving employee well-being, these efforts are also critical for sustaining care continuity and supporting the long-term viability of long-term care organizations.

## Data Availability

Data available on request from the authors.

## Abbreviations

JSS: Job Satisfaction Survey
WFCS: Work-Family Balance Scale
K10: Kessler Psychological Distress Scale
TIS: Turnover Intention Scale
IQR: Interquartile Range
CMIN/DF: Chi-square/df ratio
GFI: Goodness of Fit Index
NFI: Normed Fit Index
CFI: Comparative Fit Index
IFI: Incremental Fit Index
RMSEA: Root Mean Square Error of Approximation
VIF: Variance Inflation Factor
